# Delivery of the Placenta before the Child, in Cases of Placenta Praevia

**Published:** 1845-10-01

**Authors:** 


*Delivery of the Placenta before the Child in cases of Placenta prævia.*—A novel, and, as it seems to us, very important improvement in the management of cases of placental presentation, has lately been recommended by Dr. Redford, of Manchester, England, and Dr. Simpson, of Edinburg. It consists in the delivery of the placenta, or at least, its detatchment from the uterus, before the delivery of the child. The objects of this measure, are, first, to arrest hæmorrhage, which, according to the observations of these gentlemen and others, almost invariably ceases so soon as the separation of the placenta from the uterus is complete; and, second, to avoid the necessity of turning, which, unless there be mal-presentation or other contingent difficulties, will not be required. Certainly, if these two objects are fulfilled, there can be no doubt that preference is to be given to this new plan over that hitherto recommended, viz: turning and forcible delivery so soon as the condition of the parts will permit.—Dr. Simpson has been at pains to collect statistics showing the ratio of mortality in cases of placental presentation as reported by different authors. Out of 399 recorded cases, in 115 the result was fatal; which, as remarked by Braithwaite, is a mortality as great as that attending the malignant cholera in 1832—3, and twice as great as the average in cases of lithotomy. Dr. S. has, also, collected 141 recorded cases in which the placenta was expelled before the child. In ten of these the result was fatal; but on careful analysis, in seven or eight of these fatal cases, the fatal result had no connection with the placental detachment, or with results arising from it. He adduces this comparative view in order to substantiate the soundness of the practice which he recommends. Both writers named have pursued this practice in a considerable number of cases, and with gratifying success. The credit of originating the plan is attributed to Dr. Kinder Hood, of Manchester, who is stated to have practiced upon it as early as 1821; and since that time, many of the practitioners in Manchester have uniformly adopted it. The reader will find an abstract of the observations and reasoning of Drs. Simpson and Redford in the 11th No. of Braithwaite's retrospect. Dr. Redford, in addition, advocates the use of galvanism to excite uterine contractions when these are deficient, and, in this way, expedite the labor. He employs an electro-magnetic machine, and introduces one of the conductors into the vagina. He also recommends it in other cases where action of the uterus is desirable. We quote an account of the method of procedure in placental presentations, given by Braithwaite in his retrospect: "If the os uteri be small and rigid, we shall be obliged to wait a little for its dilatation and relaxation. As soon as possible, if the hæmorrhage be alarming, introduce the hand or a portion of it, and carefully detatch the whole of the placenta from the surface of the womb, and if the action of the organ be tolerably good, the hæmorrhage will almost immediately cease, from the mouths of the vessels being closed. If the uterus do not act energetically, it will be necessary to rupture the membranes and give secale cornutum, or, as Dr. Redford recommends, employ galvanic electricity. It will be found that even more children are saved by this means, than by the old method of detaching part of the placenta, insinuating the hand, and turning."

Cases of placenta prævia are among the most alarming which fall to the lot of the practitioner, and often put his judgment and sense of responsibility to a most painful trial. From the results of this new method, in so far as it has been pursued, we have encouragement to hope that the anxiety and danger in these cases will be materially diminished by its adoption. The facts adduced by these writers, and their experience in a limited number of cases, fully justify the trial of the plan which they propose, and we trust that a sufficient number of observations may soon be reported, to establish definitely, for or against its propriety.

